# Hydrodynamic forces on monodisperse assemblies of axisymmetric elongated particles: Orientation and voidage effects

**DOI:** 10.1002/aic.16951

**Published:** 2020-02-25

**Authors:** Sathish K. P. Sanjeevi, Johan T. Padding

**Affiliations:** ^1^ Process and Energy Department Delft University of Technology Delft The Netherlands

**Keywords:** drag, lift, and torque correlations, nonspherical particles, particle assemblies

## Abstract

We investigate the average drag, lift, and torque on static assemblies of capsule‐like particles of aspect ratio 4. The performed simulations are from Stokes flow to high Reynolds numbers (0.1 ≤ Re ≤ 1,000) at different solids volume fraction (0.1 ≤ *ɛ*_s_ ≤ 0.5). Individual particle forces as a function of the incident angle *ϕ* with respect to the average flow are scattered. However, the average particle force as a function of *ϕ* is found to be independent of mutual particle orientations for all but the highest volume fractions. On average, a sine‐squared scaling of drag and sine‐cosine scaling of lift holds for static multiparticle systems of elongated particles. For a packed bed, our findings can be utilized to compute the pressure drop with knowledge of the particle‐orientation distribution, and the average particle drag at *ϕ* = 0° and 90°. We propose closures for average forces to be used in Euler–Lagrange simulations of particles of aspect ratio 4.

## 1 INTRODUCTION

Accurate fluid‐particle drag, lift, and torque closures are required for precise Euler–Lagrangian simulations of nonspherical particles. Historically, different drag closures have been developed for assemblies of spherical particles.^1^, ^2^, ^3^ However, practical flows often involve assemblies of nonspherical particles for which there exist no closures at the moment. Even for static, monodisperse, nonspherical particle assemblies, creating the required closures is complicated due to the different possible mutual orientations of the particles. Furthermore, there is a lack of knowledge identifying the relevant parameters that can parametrize the drag, lift, and torque, which adds to the complication. Most fluidization applications involve gas–solid flows, in which case the large density ratios ensure large Stokes numbers, that is, the typical relaxation time of the solid particle velocity is large relative to the response time of the gas.^4^ It has been shown that under such conditions, it is sufficient to assume the particle configurations to be quasi‐static.^5^

Conventionally, fluidization simulations of nonspherical particles are performed by combining isolated particle drag correlations with correlations expressing the voidage effects as determined for sphere assemblies. There have been several works in the past focussing on the drag experienced by isolated nonspherical particles. Hölzer and Sommerfeld^6^ proposed a correlation for the drag coefficient *C*_D_ for nonspherical particles. The proposed correlation is a function of particle sphericity and crosswise‐sphericity, based on the projected area, which indirectly represents the particle orientation. Their proposed correlation is based on literature data of different nonspherical particles of various shapes and aspect ratios. More recently, drag, lift, and torque closures for isolated nonspherical particles have been derived based on direct numerical simulations. Zastawny et al^7^ developed drag, lift, and torque coefficients for four different nonspherical particles as a function of Reynolds number (Re) and incident angle (*ϕ*) with respect to the incoming flow. The investigated particles have aspect ratios ranging from 1.25 to 5 and Re ≤ 300. Similarly, Richter and Nikrityuk^8^ proposed fits for drag, lift, torque coefficients for cubic and ellipsoidal particles. The abovementioned literature is primarily limited to steady flow conditions. Recently, we developed drag, lift, and torque closures for three different nonspherical particles from the viscous Stokes regime upto the high Re regime of Re = 2000, involving complex, unsteady flows.^4^ In an earlier work,^9^ we reported the interesting finding that the drag coefficient *C*_D_ at different incident angles *ϕ* follows a sine‐squared scaling given by

(1) $$C_{D,\phi }=C_{D,\phi =0^{{}^{\circ}}}+\left(C_{D,\phi =90^{{}^{\circ}}}-C_{D,\phi =0^{{}^{\circ}}}\right)\sin^{2}\phi .$$

Likewise, we reported another interesting finding that the lift coefficient *C*_L_ follows sine–cosine scaling at different *ϕ* as

(2) $$C_{L,\phi }=\left(C_{D,\phi =90^{{}^{\circ}}}-C_{D,\phi =0^{{}^{\circ}}}\right)\sin\phi \cos\phi$$

for various elongated particles. The abovementioned scaling laws must be mathematically true in the Stokes regime due to linearity of the flow fields. However, their validity in the inertial regimes is primarily due to an interesting pattern of pressure distribution contributing to the drag and lift for different incident angles.^9^ In Equations 1, 2, the drag coefficients at incident angles of 0° and 90° still depend on particle shape and Reynolds number. The Reynolds number in the present work is defined as Re = | ***u***_s_|*d*_eq_/*ν*, where ***u***_s_ is the superficial flow velocity, *ν* is the kinematic viscosity of the fluid, and *d*_eq_ is the diameter of the volume‐equivalent sphere given by *d*_eq_ = (6*V*_p_/*π*)^1/3^ with *V*_p_ the particle volume.

For multiparticle systems, various literature is available to include the voidage effects, often developed through experiments and numerical simulations. One of the most widely used expressions is that of Ergun,^10^ which has been developed based on a series of packed bed experiments of different particle shapes. The only limitation of this work is that it is applicable primarily in the dense limit. Richardson and Zaki^11^ performed various sedimentation and fluidization experiments and proposed accordingly the effect of particle volume fraction on the drag. Based on the previous literature on sedimentation and packed bed experiments, Di Felice^12^ bridged the dilute and dense particulate regimes through a unified function, which also extends from low to high Re. Though the above correlations provide a good approximation, the use of such closures in Euler–Lagrangian simulations often do not represent accurate physics. This is mainly due to the inability to construct moderate solids volume fractions (*ɛ*_s_ ≈ 0.3) in experiments.

There is a growing interest to use numerical simulations to accurately develop drag closures for different Reynolds numbers Re and solids volume fractions *ɛ*_s_, albeit primarily for spheres. Initially, lattice Boltzmann method (LBM) has been the choice for simulating assemblies of spheres.^1^, ^13^, ^14^ Recently, Tenneti et al^2^ used an immersed boundary method (IBM) to develop drag closures for static assemblies of spheres for 0.01 ≤ Re ≤ 300 and 0.1 ≤ *ɛ*_s_ ≤ 0.5. They observed a deviation of 30% in the Re range from 100 to 300 with respect to the earlier work of Beetstra et al^1^ This is possible because Beetstra et al^1^ used LBM with the conventional stair‐case boundary condition to represent the sphere boundaries, for which at high Re thinner boundary layers result in larger deviations. In this work, we use a multirelaxation time (MRT) LBM for high Re flows and an interpolated bounceback scheme to much more accurately represent the particle geometry. Recently, Tang et al^3^ used an IBM based solver to create drag closures for static assemblies of spheres upto Re ≤ 1,000 and *ɛ*_s_ ≤ 0.6. We note that all mentioned works report their drag closures as the *average* drag on a collection of particles (typically a hundred to a few hundred) as a function of the *average* solids volume fraction. In reality, variations in local volume fraction and precise placement of neighboring particles will lead to a scatter in the drag per particle. However, these closures are developed for use in unresolved Euler–Lagrange (CFD‐DEM) simulations, where a typical CFD cell will be as large as the entire resolved simulation box (i.e., with a cell size typically equal to 3–6 particle lengths). It is true that in reality individual drag forces can be higher or lower than the average drag, but such detail is generally not taken into account in Euler–Lagrange simulations for computational efficiency. In general, it is assumed that the particle‐particle interactions (collisions) will lead to a rapid redistribution of particle velocities within a cell, making the average drag the most relevant factor.

There are also several disadvantages with combining an isolated nonspherical particle drag with a voidage function based on spheres. First, the assumption that the voidage effects are independent of particle shape is probably incorrect, since there exist different closures even for assemblies of polydisperse spheres.^1^, ^15^ Second, the voidage effects on lift and torque in a multiparticle system are unknown and hence are often neglected in Euler–Lagrangian simulations.^16^, ^17^ Third, using the same factor for voidage effects for all incident angles *ϕ* may hold in sufficiently dilute regimes but its validity in the dense limit is unknown. At the moment, only Li et al^18^ have discussed the drag and lift for an assembly of ellipsoidal particles. However, they have limited themselves to low Reynolds number flow (Re < 0.1), for which linearity of the flowfield automatically applies, simplifying the decomposition into drag and lift forces. Moreover, He and Tafti^19^ have discussed the drag, lift, and torque for an assembly of nonspherical particles. However, they do not propose any correlations which can be used in Euler–Lagrangian simulations. This could be due to the difficulty in identifying the dependent parameters which represent the orientation effects in nonspherical, multiparticle system adequately.

In this work, we propose and subsequently identify the important dependent parameters for static, monodisperse assemblies of axisymmetric nonspherical particles in low to high Reynolds number flow. With the identified parameters, we create closures for the average drag, lift, and torque. Our particle of interest is a capsule‐like spherocylinder of aspect ratio 4 (total length/shaft diameter). Compared to the two parameters for sphere assemblies, that is, Reynolds number Re and solids volume fraction *ɛ*_s_, we propose four additional parameters for the assembly of axisymmetric nonspherical particles. Two parameters describe the mutual orientations of the particles, namely two eigenvalues *S*_1_ and *S*_2_ of the orientation tensor, and two angle parameters *α* and *β* represent the polar and azimuthal angles of the average flow (in the coordinate frame determined by the principal directions of the order tensor). The resulting six dimensional parameter space is adequately explored and correlations are proposed accordingly. It should be noted that the fixed nature of the particles in our simulations imply that the proposed correlations are applicable for high Stokes number flows as typically experienced by Geldart D category particles.^20^ To the best of the authors' knowledge, there exists no work which parametrizes the average drag, lift, and torque for nonspherical particles in a multiparticle environment. Generally, lift and torque are ignored in large scale Euler–Lagrangian simulations. The proposed accurate drag, lift, and torque correlations enable future Euler–Lagrangian simulations to be performed with more realistic physics for these particles of aspect ratio 4.

## 2 NUMERICAL METHOD

### 2.1 Lattice Boltzmann method

In the present work, we use a D3Q19, MRT lattice Boltzmann method^21^ to simulate the fluid flow. The numerical method is adequately explained and validated in our previous works.^4^, ^9^ The evolution of particle distribution function ∣*f*〉 is computed as

(3) $$|f\left(r+e_{\alpha }\Delta t,t+\Delta t\right)〉=|f\left(r,t\right)〉-M^{-1}\hat{S}\left(|m\left(r,t\right)〉-|m^{\left(\mathrm{eq}\right)}\left(r,t\right)〉\right),$$

for position ***r*** with discrete velocities ***e***_*α*_ in directions *α* = 1, 2…, 19. Equation (3) is solved in a sequence of two steps namely collision and streaming. *M* is a 19 × 19 transformation matrix used to transform ∣*f*〉 from velocity space to moment space ∣*m*〉 with ∣*m*〉 = *M*·∣*f*〉 and the superscript (eq) in Equation (3) implies the equilibrium condition. Here, the ket vector ∣·〉 implies a column vector. The relaxation matrix $\hat{S}=M\cdot S\cdot M^{-1}$ is a 19 × 19 diagonal matrix. $\hat{S}$ utilizes different, optimally chosen relaxation rates for different moments, thereby providing better stability compared to the single‐relaxation‐time LBM scheme.^21^ The matrices *M* and $\hat{S}$ are similar to Huang et al^22^ and are given in Sanjeevi et al.^4^ The density is computed as *ρ* = ∑_*α*_ *f*_*α*_ and the momentum as *ρ* ***u*** = ∑_*α*_ *f*_*α*_ *e*_*α*_ . The relation between the kinematic viscosity of the fluid and the dimensionless relaxation time *τ* is $\nu =c_{s}^{2}\left(\tau -1/2\right)\Delta t$, and the pressure *p* is related to the density by $p=\rho c_{s}^{2}$, where *c*_s_ is the speed of sound. A linearly interpolated bounce back scheme^23^, ^24^ is used to accurately consider the curved geometry of the particle, as opposed to the traditional stair‐case bounce back boundary condition. The flow is driven by a body force ***g*** and the simulated domain is periodic in all three directions. The use of the interpolated bounce back scheme within a periodic domain results in a slow mass leakage/gain in the system. Accordingly, the mass is corrected using a case 3 type correction described in Sanjeevi et al.^25^ The results for the multiparticle system are validated in subsequent sections.

The ratio of *d*_eq_/*d*_min_ equals 1.765 for the considered spherocylinder of aspect ratio 4, where *d*_min_ implies diameter of the cylinder. The simulation parameters used in our LBM simulations are summarized in Table 1. Specifically, it can observed that a good particle resolution (*d*_eq_) is maintained for different Re. Further with increasing *ɛ*_s_, the *d*_eq_ is increased accordingly to resolve increased velocity gradients at high *ɛ*_s_. All LBM simulations have cubic domain, each with 200 particles unless otherwise specified. At least two independent simulations are performed for each Re and *ɛ*_s_ and the details of independent number of simulations are discussed later (see Figure 13).

**Table 1:** Details of the simulation parameters used in our simulations in LB units

Re	*L*_D_	*d*_eq_	*ν*
0.1 ≤ Re ≤ 10	288	28.36–48.5	1.3/3
10 < Re ≤ 100	576	56.72–97.0	0.1–0.08/3
300	576	56.72–97.0	0.04/3
600	576	56.72–97.0	0.015/3
1,000	768	75.63–129.3	0.01/3

*Note: L*_D_ denotes the side length of the cubic domain. The range of *d*_eq_ specified is respectively for 0.1 ≤ *ɛ*_s_ ≤ 0.5.

### 2.2 Flow control

In order to perform a simulation for a specific Re, it is required to control the superficial flow velocity ***u***_s_ by applying a body force ***g***. The relationship between the superficial velocity and the average interstitial flow velocity ***u***_avg_ is given by ***u***_s_ = (1 − *ɛ*_s_) ***u***_avg_. Due to the nonspherical nature of the particles, the sum of lift forces is often non‐zero, and the resultant direction of ***u***_s_ can be different from the direction of ***g***. This necessitates the need to control both direction and magnitude of the body force. Initially, the fluid is at rest with both ***u***_s_ and ***g*** zero. The flow is slowly ramped up by increasing ***g*** until the desired ***u***_s_ is achieved. For each timestep, the updated gravity ***g***_new_ is computed as

(4) $$g_{\mathrm{new}}=g_{\text{prev}}+\frac{\left(u_{s,\mathrm{ref}}-u_{s,\text{prev}}\right)}{K_{p}^{2}}\Delta t,$$

where ***g***_prev_ is the gravity from the previous timestep, ***u***_s, ref_ is the desired superficial velocity, and ***u***_s, prev_ is the superficial velocity from the previous timestep. *K*_p_ is a time constant which controls the system response rate. The stopping criterion for the simulations is when the system ***u***_s_ reaches 99.9% of the reference setpoint.

## 3 SIMULATION SETUP

### 3.1 Orientation tensor

In this section, we briefly explain the characterization of mutual orientations in an assembly of axisymmetric nonspherical particles with an orientation tensor. We subsequently explain the use of a Maier‐Saupe potential to achieve the desired particle configurations through Monte‐Carlo simulations.

To describe the orientation of a single axisymmetric particle, the azimuthal and polar angles are sufficient. For a multiparticle configuration, it is important to parametrize the mutual orientations of the particles, with the least number of parameters. For this, we propose to use the orientation tensor *S*, also known in literature as the nematic order tensor,^18^, ^26^, ^27^ defined as

(5) $$S=〈{\mathrm{pp}}^{T}〉.$$

**Figure 1:**
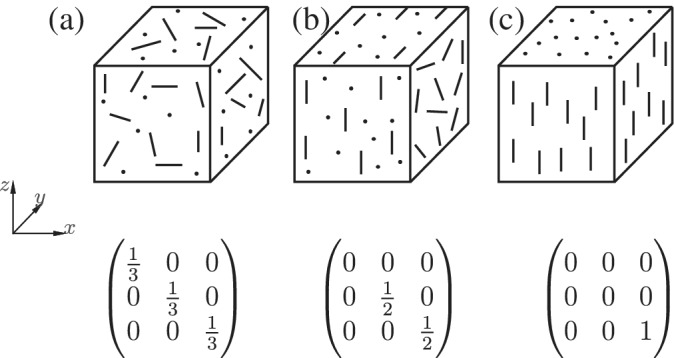
Different particle configurations and their orientation tensors: (a) random, (b) planar random, and (c) unidirectional (nematic) configuration

Here, ***p*** is the unit orientation vector of a particle along the axis of symmetry. The three eigenvalues (which we order as *S*_1_, *S*_2_, *S*_3_ from small to large) characterize the type of mutual alignment, as shown in Figure 1. The corresponding three eigenvectors define the principal directions of mutual particle alignment.

Because the trace of *S* is 1, only two eigenvalues are sufficient to specify the amount of randomness, planar random (biaxial), or unidirectional (nematic) order. Note that the tensor *S* is insensitive to an orientation ***p*** or − ***p*** of particles. In other words, the tensor captures essentially the mutual alignment of particles irrespective of particles oriented in positive or negative direction. Figure 1a shows a completely random configuration with *S*_1_ = *S*_2_ = *S*_3_ = 1/3. Figure 1b shows a planar random configuration with particles primarily confined to planes (in this example with random orientations in planes normal to the *x*‐direction) resulting in *S*_1_ = 0, *S*_2_ = *S*_3_ = 1/2, and similarly a unidirectional (nematic, in this example in the *z*‐direction) configuration in Figure 1c with *S*_1_ = *S*_2_ = 0, *S*_3_ = 1. In practical conditions, particles can exhibit complex configurations in between these extremes but can be adequately described by two eigenvalues *S*_1_ and *S*_2_. Regarding the unidirectional case, we consider only nematic configurations but not smectic because ordering of both positions and orientations is rare in fluidization conditions.

The above metrics can be used to describe the particle configuration. However, due to the nonsphericity of the particles, the flow orientation with respect to the principal directions of the particle orientations is also important. This results in two parameters, namely the polar angle (*α*) and azimuthal angle (*β*) of the average flow velocity vector with respect to the space spanned by the three eigenvectors of the orientation tensor. In summary, the parameter space to be explored for our flow problem has six parameters, namely Reynolds number Re, solids volume fraction *ɛ*_s_, two particle configuration parameters *S*_1_, *S*_2_ and two angles *α* and *β* describing the mean flow orientation with respect to the configuration.

### 3.2 Generation of biased particle configurations

The generation of nonoverlapping configurations of the particles in a periodic domain is required as an input for the flow simulations. It is also required to generate configurations of particles with a prescribed orientation tensor, which adds further complexity. In this section, we briefly describe the Monte‐Carlo simulation algorithm for generating configuration of nonoverlapping particles and the use of a Maier‐Saupe potential^28^ to bias the system to the required orientation tensor.

As the particles are spherocylindrical in shape, a simple way to detect overlap is to find the minimum distance between two line segments. We define the line segment as the line connecting the centres of the two spheres at the extremes of the spherocylinder. If the distance between two line segments is less than the particle diameter (i.e., sum of the radii of two interacting particles), then the spherocylinders overlap. A fast algorithm is used to measure the shortest distance between the line segments.^29^

Using the above overlap detection algorithm, randomly picked particles are randomly translated in small (compared to the particle diameter) steps and rotated by a small angle around a randomly chosen axis. Because our system is always below the threshold for a spontaneous nematic order transition, this procedure results in a random configuration after many iterations. If a prescribed amount of mutual orientation is required, besides the requirement of no overlap, a Monte‐Carlo procedure is applied to decide whether to accept or reject a new orientation of a particle. In detail, we choose a principal director ***n***, which is a reference vector to which the particles are biased to align with or against (depending on the sign of the magnitude *A* of the Maier‐Saupe potential). In the Monte‐Carlo approach a new orientation ***p***_new_ of a randomly picked particle, having current orientation ***p***_curr_, is accepted or rejected based on the following criteria:

(6) $$p_{\mathrm{new}}=\left\{\begin{array}{ll} p_{\mathrm{new}}, & \text{if}\;\Delta E<0\\ p_{\mathrm{new}}, & \text{if}\;\Delta E\geq 0\;\text{and}\;U\left(\left[0,1\right]\right)<\exp\left(-\Delta E\right)\\ p_{\text{curr}}, & \text{otherwise} \end{array}\right.$$

(7) $$\;\text{where}\;\Delta E=A\left[{\left(p_{\mathrm{new}}\cdot n\right)}^{2}-{\left(p_{\text{curr}}\cdot n\right)}^{2}\right].$$

Here, Δ*E* is the change in Maier‐Saupe potential and *U*([0, 1]) is a random number uniformly distributed between 0 and 1. Of the three conditions in Equation (6), it is clear that the first condition accepts the new orientation if it leads to a lower Maier‐Saupe potential. Without the second condition, the system would approach toward an ideal mutual orientation (such as perfect parallel or perfectly perpendicular particles w.r.t. the principal director, depending on the sign of *A*) when the Monte‐Carlo simulation is run for a sufficiently long time. With the second condition, however, increases in the Maier‐Saupe potential are also accepted with a certain probability less than 1 (the larger the increase the potential, the smaller the probability of acceptance). After sufficiently long time, a balance is found between the random particle reorientations and particle orientation ordering by the Maier‐Saupe potential, leading to a degree of randomness that can be controlled by the magnitude of the user specified constant *A*. A bias toward planar random configuration is achieved when *A* > 0, with more particles oriented in planes perpendicular to the director ***n***. A bias toward unidirectional (nematic) configuration is achieved when *A* < 0, with more particles oriented along the direction of ***n***.

**Figure 2:**
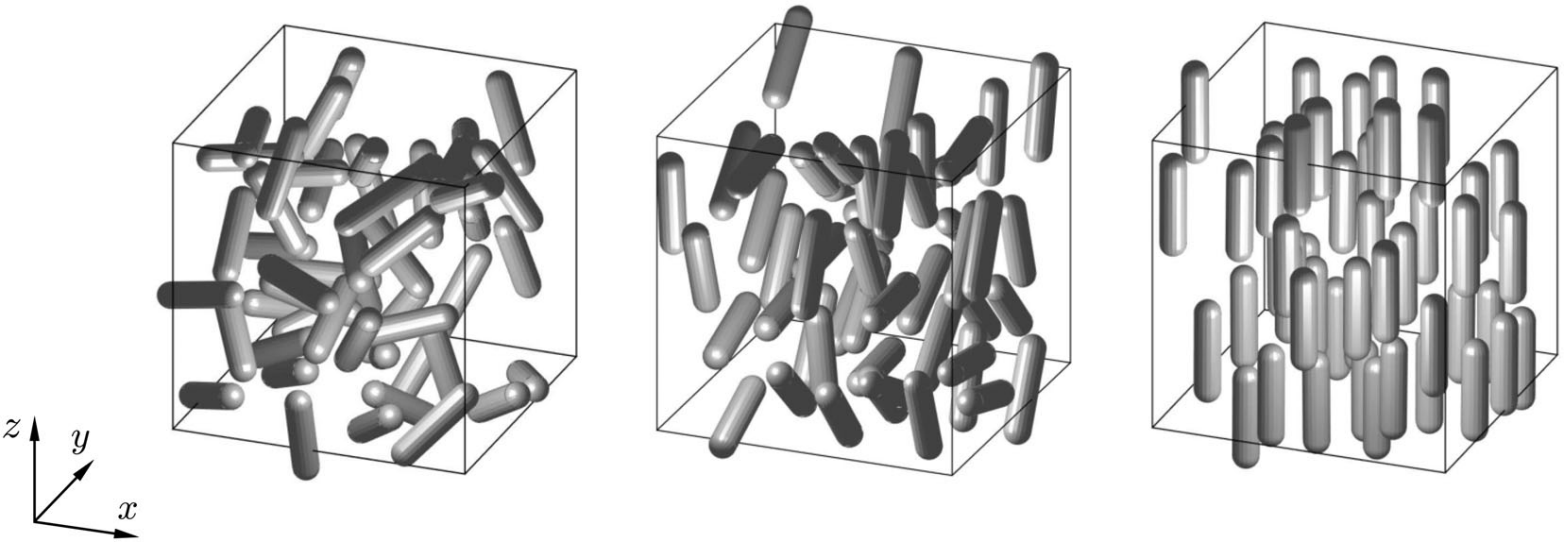
Different configurations of nonspherical particles generated using the Monte‐Carlo simulations: (a) Random configuration without the use of Maier‐Saupe potential, (b) planar random, and (c) unidirectional configuration generated using the Maier‐Saupe potential. For better clarity, the shown examples have only 50 particles. The actual simulations involve 200 particles

With the mentioned strategy, any configuration in‐between the ideal cases shown in Figure 1 can be achieved. Some sample configurations generated using the abovementioned algorithm are shown in Figure 2. For simplicity, the eigenvectors of the orientation tensor *S* are considered as aligned with the Cartesian coordinate system in Figure 2. The shown configurations are respectively equivalent to Figure 1. For better clarity, the shown configuration has only 50 particles and the solids volume fraction *ɛ*_s_ is 0.1. The actual flow simulations have 200 particles and are performed for various *ɛ*_s_.

**Figure 3:**
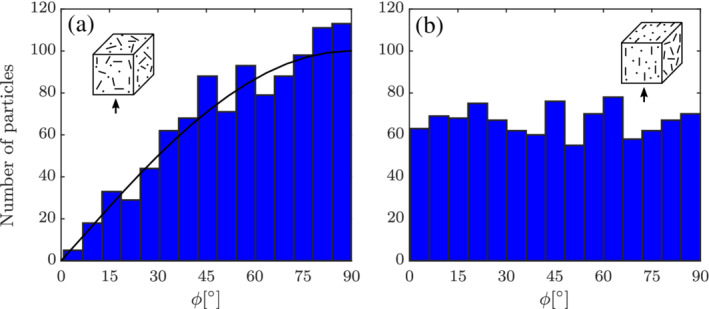
Histogram of particles with different incident angles *ϕ* with respect to the flow vector (indicated by an arrow) for (a) random and (b) planar random configuration. The shown example has 1,000 particles. It should be noted that the *ϕ* distribution for a random configuration will always scale as sin*ϕ* (solid black curve) irrespective of the flow direction [Color figure can be viewed at wileyonlinelibrary.com]

A common intuition may be that a random configuration would result in particles with evenly distributed values of the incident angle *ϕ*. However, for a random configuration, the available number of particles at different *ϕ* are not uniform, as shown in Figure 3a. This is due to the higher probability to find particles at an angle *ϕ* near 90° because the Jacobian for a spherical coordinate system scales as sin*ϕ* . Therefore, the disadvantage for a random configuration is that there are actually few data points at *ϕ* = 0° to create angle‐dependent closures. On the contrary, the planar configuration with the planes parallel to the flow direction results in even particle distributions, as shown in Figure 3b. This information is considered while we generate configurations for the flow simulations.

### 3.3 Forces and torques acting on a particle

For an assembly of particles, different definitions are used to report the forces.^1^, ^2^, ^3^ To ensure consistency, it is important to know the form of the reported results. For a packed bed of particles in a flow induced by a macroscopic pressure gradient ∇*P*, each particle of volume *V*_p_ experiences a resulting force ***F*** due to the flow alone, and a buoyancy force ***F***_b_ = −*V*_p_∇*P* due to the pressure gradient. For such a case, the total fluid‐to‐particle force ***F***_*f* → *p*_ acting on a particle is

(8) $$F_{f\to p}=F+F_{b}.$$

Given *N* particles with each of volume *V*_p_ and total volume of the system *V*, the solids volume fraction is given by *ɛ*_s_ = *NV*_p_/*V*. Further, the relationship between ***F*** and ***F***_*f* → *p*_ is given by^3^

(9) $$F=F_{f\to p}\left(1-{ɛ}_{s}\right).$$

In this work, we report the forces ***F*** due to the flow and not ***F***_*f* → *p*_ . Note that in some simulation packages ***F***_*f* → *p*_ is needed, in which case the correlations we report in this work should be divided by (1 − *ɛ*_s_). The effects of buoyancy on torques are unknown and hence the reported torques ***T*** are also as they are determined from the simulations. We normalize the force and torque with the Stokes drag and torque of a volume‐equivalent sphere:

(10) $$F_{\mathrm{norm}}=\frac{F}{6\pi \mu R_{\mathrm{eq}}|u_{s}|},\;\text{and}$$

(11) $$T_{\mathrm{norm}}=\frac{T}{8\pi \mu R_{\mathrm{eq}}^{2}|u_{s}|}.$$

Here, *μ* is the dynamic viscosity and *R*_eq_ is the radius of the volume equivalent sphere. The Stokes torque that we used is based on the torque experienced by a rotating sphere in still fluid.^30^

Let ***p*** be the normalized orientation vector of the considered particle. The local coordinate system for each particle is defined as

(12) $${\hat{e}}_{1}=\frac{u_{s}}{|u_{s}|},$$

(13) $${\hat{e}}_{2}=\frac{{\hat{e}}_{1}\times p}{|{\hat{e}}_{1}\times p|}\mathrm{sign}\left({\hat{e}}_{1}\cdot p\right),\;\text{and}$$

(14) $${\hat{e}}_{3}={\hat{e}}_{1}\times {\hat{e}}_{2}.$$

**Figure 4:**
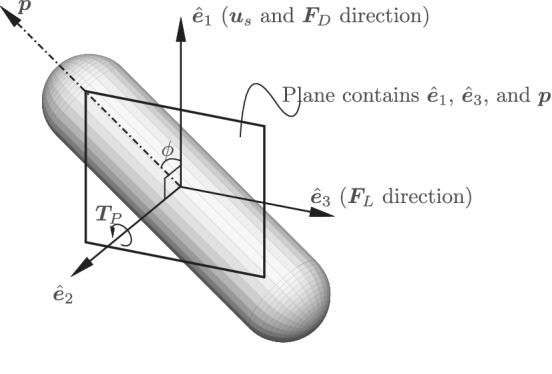
The local coordinate system of a particle. ***u***_s_ and ***F***_D_ act along ${\hat{e}}_{1}$, ***F***_L_ along ${\hat{e}}_{3}$ and ***T***_P_ about the ${\hat{e}}_{2}$ axis

The above defined axes are accordingly illustrated in Figure 4. The incident angle *ϕ* a particle makes with respect to the incoming flow is given by $\phi =\cos^{-1}\left(\left|{\hat{e}}_{1}\cdot p\right|\right)$. We also compute the average forces and torques for different *ϕ* intervals. Due to the finite number of measurements in these intervals, there is an error on the mean $\bar{x}$ of any property *x*. We use the standard error on the mean $\sigma_{\bar{x}}$ for the errorbars, computed as

(15) $$\sigma_{\bar{x}}=\sigma /\sqrt{n}.$$

Here, *σ* is the standard deviation of the corresponding variable *x* and *n* is the number of data points within the given *ϕ* interval. Throughout this work, we use overbar (^–^) to denote arithmetic averages and boldface to denote vectors.

The normalized drag *F*_D_ and lift *F*_L_ can be computed from ***F***_norm_ as

(16) $$F_{D}=F_{1}=F_{\mathrm{norm}}\cdot {\hat{e}}_{1},$$

(17) $$F_{2}=F_{\mathrm{norm}}\cdot {\hat{e}}_{2},\;\text{and}$$

(18) $$F_{L}=F_{3}=F_{\mathrm{norm}}\cdot {\hat{e}}_{3}.$$

**Figure 5:**
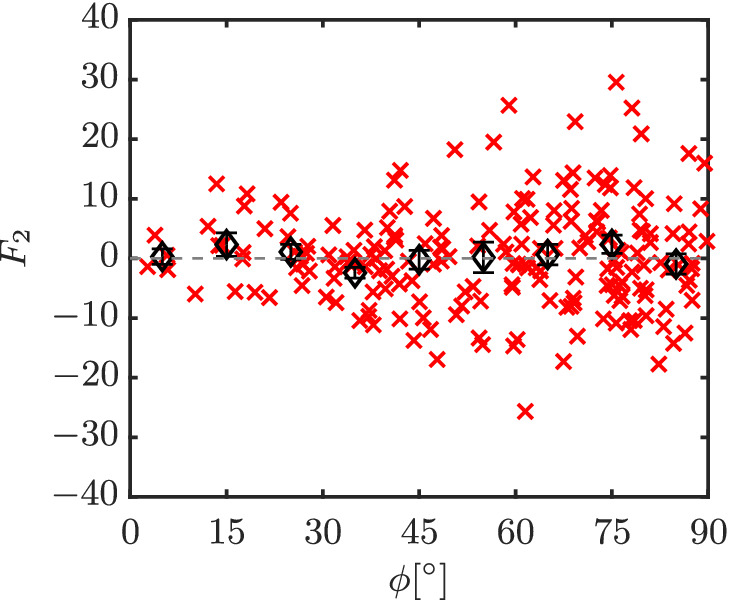
Lateral force *F*_2_ distribution for different particles (**×**) with averages at regular *ϕ* intervals (*◊*) in a random configuration at Re = 100 and *ɛ*_s_ = 0.3. The error bars indicate the standard error on the mean for each *ϕ* interval [Color figure can be viewed at wileyonlinelibrary.com]

Since the reported forces are without buoyancy effects, the (1 − *ɛ*_s_) term must be considered accordingly for both drag and lift while performing Euler–Lagrangian simulations. Due to the influence of neighboring particles, the lateral force *F*_2_ for each individual particle may not be equal zero, as shown in Figure 5 (Re = 100 and *ɛ*_s_ = 0.3). However, due to symmetry, the average *F*_2_ does equal zero. Therefore, *F*_2_ is not considered in our further discussion. The torques about the above defined axes are

(19) $$T_{1}=T_{\mathrm{norm}}\cdot {\hat{e}}_{1},$$

(20) $$T_{P}=T_{2}=T_{\mathrm{norm}}\cdot {\hat{e}}_{2},\;\text{and}$$

(21) $$T_{3}=T_{\mathrm{norm}}\cdot {\hat{e}}_{3}.$$

**Figure 6:**
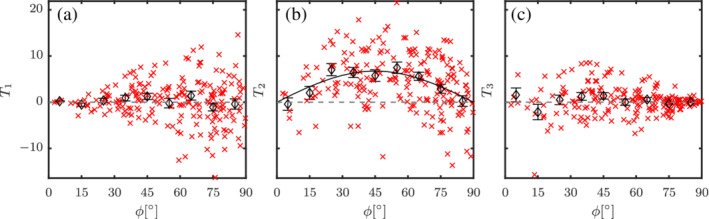
Torques (a) *T*_1_, (b) *T*_2_, and (c) *T*_3_ distribution for different particles (**×**) with averages at regular *ϕ* intervals (*◊*) in a random configuration at Re = 100 and *ɛ*_s_ = 0.3. Due to flow symmetry, the average *T*_1_ and *T*_3_ acting on particles are statistically zero. However, the pitching torque *T*_P_ (or *T*_2_) scales proportional to sin*ϕ*cos*ϕ* (solid black line). The error bars indicate the standard error on the mean for each *ϕ* interval [Color figure can be viewed at wileyonlinelibrary.com]

Here, *T*_P_ is the pitching torque acting on a particle. We show the three different torques for a flow through a random particle configuration at Re = 100 and *ɛ*_s_ = 0.3 in Figure 6. It can be observed that *T*_1_ and *T*_3_, though having some non‐zero values, are statistically zero on average due to symmetry. The non‐zero values are primarily due to hydrodynamic interactions with other particles. Only the average pitching torque *T*_P_ (or *T*_2_) remains non‐zero for different *ϕ* and varies as sin*ϕ*cos*ϕ* . Though individual particles experience non‐zero *T*_1_ and *T*_3_, they become zero at *ϕ* = 0° and *ϕ* = 90°, respectively, where the axis of symmetry of the particle coincides with the measured axis for torque. This implies that the hydrodynamic interaction of particles does not induce a torque (or a spin) about the axis of symmetry of the particle.

### 3.4 Validation

**Figure 7:**
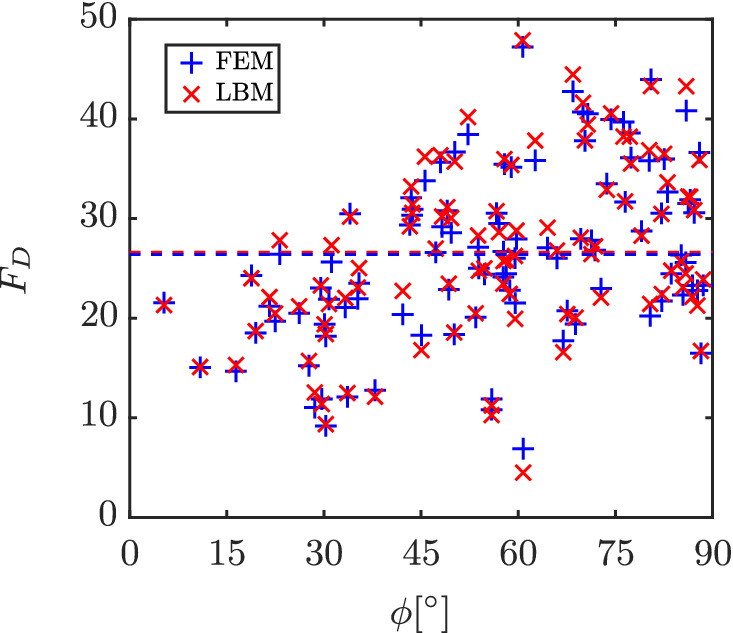
*F*_D_ obtained for individual particles in a random configuration from the LBM solver against an incompressible flow FEM solver for Re = 100 and *ɛ*_s_ = 0.3. The dashed lines in respective colors indicate the domain averages from the respective solvers [Color figure can be viewed at wileyonlinelibrary.com]

Sufficient validation has been done for our LBM code in the past for flow around isolated particles.^4^, ^9^ For a multiparticle configuration, we have chosen flow around a random assembly of 100 particles at Re = 100 and *ɛ*_s_ = 0.3 and measure the *F*_D_ experienced by the individual particles. The LBM results are compared with results from COMSOL Multiphysics, a body‐fitted, unstructured mesh based incompressible flow FEM solver. The simulated LBM domain is of size 360^3^. The volume equivalent sphere diameter is *d*_eq_ = 64.4 lattice cells. The superficial velocity *u*_s_ is 0.0414 and the kinematic viscosity *ν* is 0.08/3 in lattice units. The FEM solver domain is made of 2.1 million elements. The resulting drag forces are shown in Figure 7. A good agreement between LBM and FEM results can be observed. The average *F*_D_ experienced by all particles in LBM and FEM solvers are 26.6 and 26.4, respectively. Also, a good match in *F*_D_ values for individual particles at different *ϕ* can be observed. We note that in all simulations the flow velocities remained sufficiently low to avoid finite compressibility effects. In the worst case, the local Mach number was Ma = *v*/*c*_s_ = 0.2 in a few regions in the simulation box. Even under those worst circumstances, the relative density variations were observed to be at most 2%, which is why our results can be considered to be in the incompressible limit.

### 3.5 Tests of configuration independence

Given a six‐dimensional parameter space, exploring each dimension with approximately five simulations, results in a massive 5^6^ = 15,625 simulations. Furthermore, closures must be created for drag, lift, and torque as a function of this six‐dimensional space. Before proceeding with these simulations, we tried to identify if there are any independent parameters specifically related to the mutual orientation of particles. In this section, we will show that the average hydrodynamic force on a nonspherical particle is independent of the mutual orientation of the particles themselves. This configuration independence removes the configuration parameters *S*_1_, *S*_2_ and flow angle parameters *α* and *β* from the parameter space to be explored. We find that, when averaged over a number of particles, the only dependence that the particles exhibit regarding orientation is the particle's incident angle *ϕ* as in flow around single particles. Effectively, we will show that the average force depends only on the Reynolds number Re, solids volume fraction *ɛ*_s_ and the incident angle *ϕ* of individual particles with respect to the flow direction.

**Figure 8:**
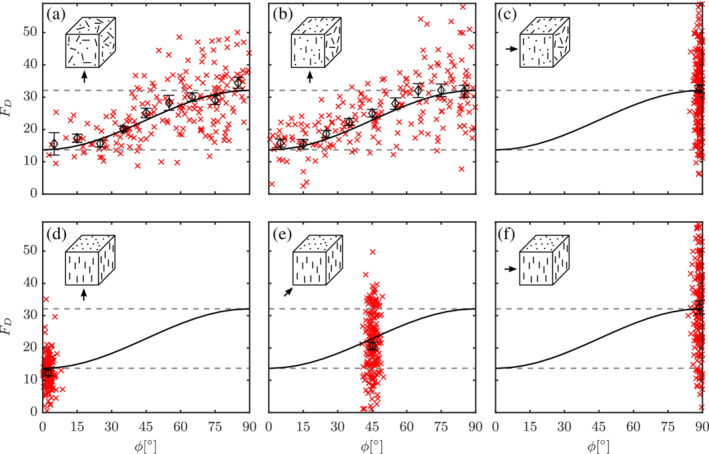
Configuration independence phenomenon at Re = 100 and *ɛ*_s_ = 0.3 for different configurations with different flow directions (arrow indicated). *F*_D_ distribution for different particles (**×**) with averages at regular *ϕ* intervals (*◊*). (a) Random configuration, planar random configuration with flow (b) parallel, and (c) perpendicular to the plane, unidirectional configuration with flow at (d) 0°, (e) 45°, and (f) 90° with respect to the principal configuration director. The solid black line indicates the sin^2^ *ϕ* scaling. The error bars indicate the standard error on the mean for each *ϕ* interval [Color figure can be viewed at wileyonlinelibrary.com]

In the extremely dilute regimes, that is, ɛ_s_ → 0, it is already shown that there exists a sine‐squared scaling of drag for elongated nonspherical particles.^4^, ^9^ In this section, we discuss the results of flow around different configurations at an intermediate solids volume fraction of *ɛ*_s_ = 0.3. Results of different configurations (in respective plot insets) at an intermediate Re = 100 are shown in Figure 8 such as fully random, planar random with flows parallel and perpendicular to the planes, and unidirectional configurations with principal directors at different angles. Though there exists scatter in the measured *F*_D_ on individual particles, it can be observed that the average drag ${\bar{F}}_{D}$ for different *ϕ* intervals scales similar to sine‐squared scaling as in our earlier works of isolated particles. In other words, the average drag ${\bar{F}}_{D}$ at any *ϕ* can be computed as

(22) $${\bar{F}}_{D,\phi }={\bar{F}}_{D,\phi =0^{{}^{\circ}}}+\left({\bar{F}}_{D,\phi =90^{{}^{\circ}}}-{\bar{F}}_{D,\phi =0^{{}^{\circ}}}\right)\sin^{2}\phi .$$

**Figure 9:**
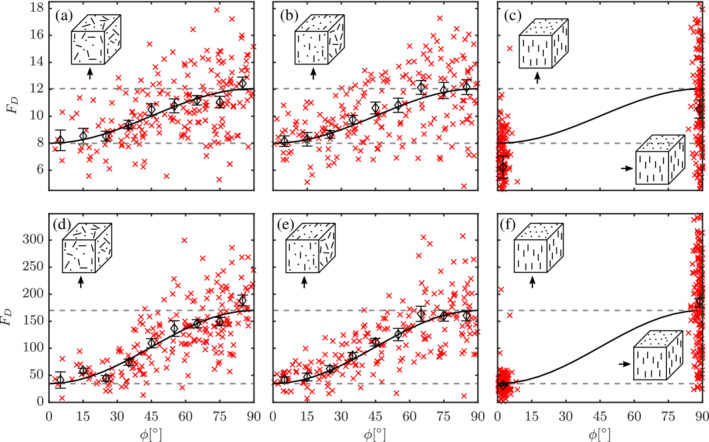
Configuration independence phenomenon at moderate solids fraction *ɛ*_s_ = 0.3 for (a–c) Re = 0.1 (low Re) and (d–f) Re = 1,000 (high Re) for different configurations and different flow directions (arrow indicated). *F*_D_ distribution for different particles (**×**) with averages at regular *ϕ* intervals (*◊*). (a, d) Random configuration, (b, e) planar random configuration with flow parallel to the plane, (c, f) combined results of unidirectional configuration with flow 0° and 90° with respect to the principal configuration director. The solid black line indicates the sin^2^ *ϕ* scaling. The error bars indicate the standard error on the mean for each *ϕ* interval [Color figure can be viewed at wileyonlinelibrary.com]

It is important to note that the *same* values for average ${\bar{F}}_{D,\phi =0^{{}^{\circ}}}$ and ${\bar{F}}_{D,\phi =90^{{}^{\circ}}}$ emerge for all configurations: the solid lines in Figure 8 are obtained as a single fit to the data from *all* configurations investigated at a certain *ɛ*_s_. Likewise, we also show that the scaling phenomenon extends to both Stokes and high Re regimes in Figure 9. With the sine‐squared scaling behavior (or the configuration independence) identified at *ɛ*_s_ = 0 and *ɛ*_s_ = 0.3, it can be inferred that the scaling is safely applicable in the region 0 ≤ *ɛ*_s_ ≤ 0.3. We have verified the same at *ɛ*_s_ = 0.1 and the results are not shown here for brevity. Though we observe the results are dependent on only three parameters, namely Re, *ɛ*_s_, and *ϕ*, the simulation needs to be set up for only two parameters, namely Re and *ɛ*_s_. With a sufficiently random configuration, the system involves different particle orientations covering all *ϕ*. A caveat with a random configuration is that there are always very few particles near *ϕ* = 0°, as shown in Figure 3. Therefore, biased random configurations with more particles at *ϕ* = 0° are created and at least two simulations are performed for better statistics.

**Figure 10:**
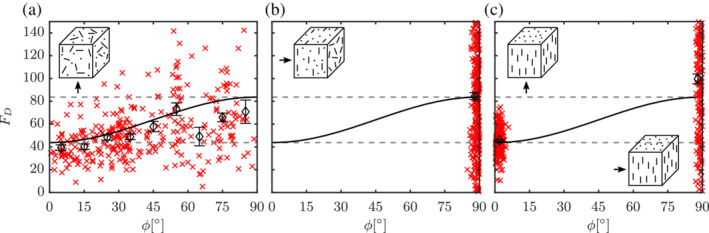
Configuration independence phenomenon at dense solids fraction *ɛ*_s_ = 0.5 for Re = 100 for different configurations and different flow directions (arrow indicated). *F*_D_ distribution for different particles (**×**) with averages at regular *ϕ* intervals (*◊*). The error bars indicate the standard error on the mean for each *ϕ* interval [Color figure can be viewed at wileyonlinelibrary.com]

We also observe the configuration independence phenomenon at *ɛ*_s_ = 0.4. The criterion considered to declare configuration independence phenomena is that the average drag results in a given *ϕ* range of different configurations are within 10% deviation. In almost all cases, the deviations are within ±5%. However, in a dense case with *ɛ*_s_ = 0.5, several more factors such as the mutual orientations and relative positions of particles. influence the results. The *F*_D_ distribution for such dense configurations at Re = 100 and *ɛ*_s_ = 0.5 are given in Figure 10. Although these results can be predominantly parametrized by Re, *ɛ*_s_, and *ϕ*, the influence of the additional parameters cannot be ignored. Therefore, specific cases of *ɛ*_s_ = 0.5 are performed with more simulations for better statistics.

**Figure 11:**
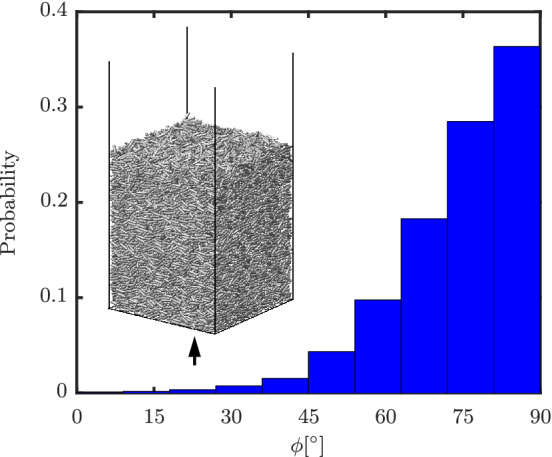
Histogram of incident angle *ϕ* for a packed bed with 30,000 particles of aspect ratio 4. The arrow indicates the flow direction [Color figure can be viewed at wileyonlinelibrary.com]

For a practical fluidization or other relevant gas–solid flow simulation, the densest configuration is most likely to occur when the particles are at bottom or at rest (e.g., before the start of fluidization). In such a dense condition, the particle configuration itself is dependent on the wall geometry. For a typical bed configuration with a flat wall at the bottom, the particles also roughly align in planes parallel to the bottom wall, that is, a planar random configuration. Pournin et al^31^ observed the same for particles poured freely from the top. Similarly, we also observe the same for a bed containing freely poured particles settled under gravity (*ɛ*_s_ = 0.54), as shown in Figure 11. The bed contains 30,000 particles and it can be observed that roughly 2/3 of all particles are in the range *ϕ* = 70–90° confirming our hypothesis. Given such criteria, the most relevant regime would be to generate an accurate fit for average ${\bar{F}}_{D,\phi =90^{{}^{\circ}}}$ at high *ɛ*_s_, which would help to predict minimum fluidization velocity of the bed accurately.

It should also be noted that with increasing aspect ratio of elongated particles, the maximum *ɛ*_s_ decreases for a packed bed.^32^ This is because the locking phenomenon is stronger with high aspect ratio particles. Unless the particles are packed with their orientations aligned, the decrease in peak *ɛ*_s_ for high aspect ratio elongated particles is unavoidable. Also, practical applications as shown in Figure 11 do not allow such long range ordering. A decreasing peak *ɛ*_s_ implies that the configuration independence phenomenon will be very applicable.

With the observed sine‐squared drag scaling, the pressure drop across a packed bed can be determined with the knowledge of the *ϕ* distribution alone. For example, for a truly random orientation, the probability of having an angle *ϕ* between rod orientation and average flow orientation scales as sin(*ϕ*). Together with the sin^2^(*ϕ*) dependence of the drag on orientation, this predicts an average drag (and associated pressure drop) equal to $\frac{2}{3}{\bar{F}}_{D,\phi =90^{{}^{\circ}}}+\frac{1}{3}{\bar{F}}_{D,\phi =0^{{}^{\circ}}}$ at the given Re and *ɛ*_s_.

In the subsequent sections, we will show that in the dilute and intermediate *ɛ*_s_ regimes, the influence of *ɛ*_s_ is nearly shape independent. This implies that the drag on isolated nonspherical particles can be combined with sphere‐based multiparticle correlations for the voidage effect to mimic flow around assemblies of nonspherical particles upto intermediate *ɛ*_s_.

### 3.6 Explored regimes

**Figure 12:**
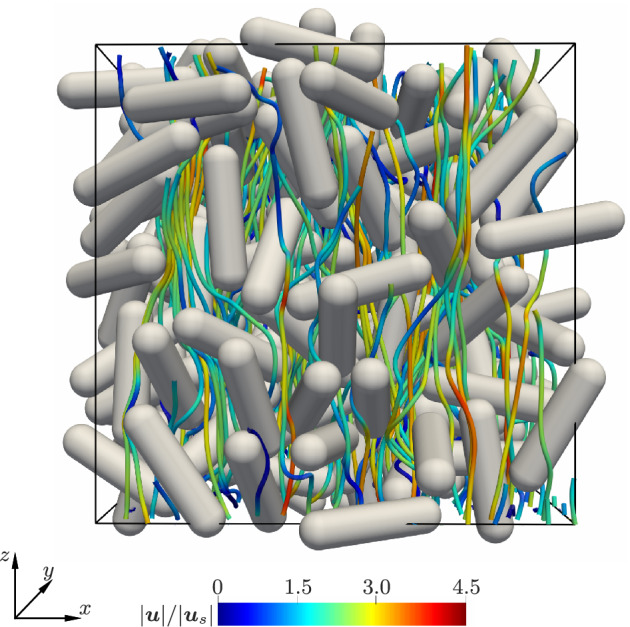
Flow streamlines for a random configuration of aspect ratio 4 particles at Re = 100 and *ɛ*_s_ = 0.3 [Color figure can be viewed at wileyonlinelibrary.com]

**Figure 13:**
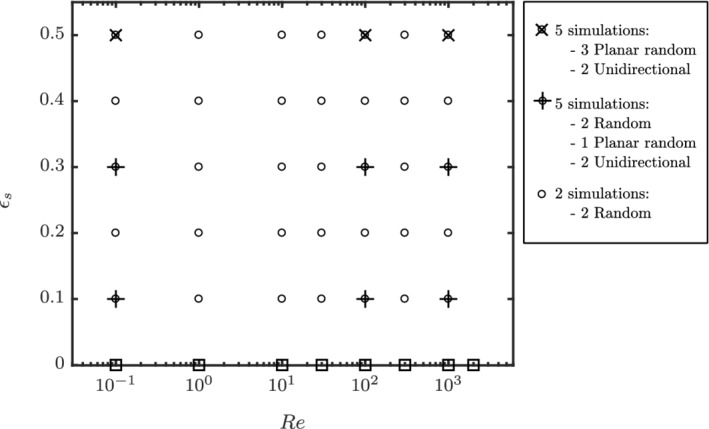
Regime map containing the explored parameter space in the current work (∘) and our previous work^9^ (□). +, × indicate the regimes with extra simulations and tested for configuration independence

In this section, we briefly explain the regimes explored in the current work and also explain the number of independent simulations performed per regime tested. An example of the flow stream lines for a random configuration at Re = 100 and *ɛ*_s_ = 0.3 is shown in Figure 12. Until solids volume fractions of *ɛ*_s_ = 0.35, the generation of randomly orientation configurations is possible, as experienced by He and Tafti^19^ for prolate spheroids of aspect ratio 2.5. In our case, we are able to achieve random configurations upto *ɛ*_s_ = 0.4. However, for denser configurations, it is difficult to generate a truly random configuration. For dense configurations of *ɛ*_s_ = 0.5, the particles have a natural tendency to orient to planar random or unidirectional orientation configurations. A truly random configuration with a finite number of particles, at such solids volume fraction, is not possible. This is due to a strong orientation bias imposed by neighboring particles due to lack of interparticle space. The explored flow regimes are indicated in Figure 13. Overall, at least two simulations are performed for the explored regimes. However, for specific cases of dilute and intermediate *ɛ*_s_, we performed five simulations with 2 random, 1 planar random with flow aligned to the plane and 2 unidirectional configurations with flow parallel and perpendicular to the principal director. For solids fraction *ɛ*_s_ = 0.5, 3 planar random configurations with flows aligned to the plane and 2 unidirectional configurations with flows parallel and perpendicular to the principal director are performed. For cases with more simulations, the results are accordingly weighted while making the fits.

## 4 RESULTS

### 4.1 Drag

With sine‐squared scaling valid for all particle mutual orientations, as shown in the previous section, the average drag experienced by a particle in a multiparticle system can be explained by the Equation (22) involving only the average drag experienced at *ϕ* = 0° and *ϕ* = 90°. Therefore, we propose to generate fits for average ${\bar{F}}_{D,\phi =0^{{}^{\circ}}}$ and ${\bar{F}}_{D,\phi =90^{{}^{\circ}}}$ as a function of Re and *ɛ*_s_ as

(23) F¯DReɛs=Fd,isol⋅1−ɛs2+Fɛs+FRe,ɛs.

The corresponding terms are as follows:

(24) Fd,isolRe=Cd,isolRe24,

(25) $$F_{{ɛ}_{s}}\left({ɛ}_{s}\right)=a\sqrt{{ɛ}_{s}}{\left(1-{ɛ}_{s}\right)}^{2}+\frac{b{ɛ}_{s}}{{\left(1-{ɛ}_{s}\right)}^{2}},\;\text{and}\;$$

(26) FRe,ɛsReɛs=Recɛsde1−ɛs+fɛs31−ɛs+gɛs1−ɛs2Re.

Here, *C*_d, isol_ is the isolated particle drag at given Re as detailed in Reference 4 for the considered particle (fibre) for both *ϕ* = 0° and *ϕ* = 90°. The coefficients in Equations 25, 26 for both average ${\bar{F}}_{D,\phi =0^{{}^{\circ}}}$ and ${\bar{F}}_{D,\phi =90^{{}^{\circ}}}$ are given in Table 2. The average absolute deviation of the fits and simulation data are 3.5 and 2% for ${\bar{F}}_{D,\phi =0^{{}^{\circ}}}$ and ${\bar{F}}_{D,\phi =90^{{}^{\circ}}}$, respectively.

**Table 2:** Coefficients of the fits for average ${\bar{F}}_{D}$ and ${\bar{F}}_{L}$

Coefficients	${\bar{F}}_{D}$		*F*_L, mag_
	*ϕ* = 0°	*ϕ* = 90°	
*a*	2	3	0.85
*b*	11.3	17.2	5.4
*c*	0.69	0.79	0.97
*d*	0.77	3	0.75
*e*	0.42	11.12	−0.92
*f*	4.84	11.12	2.66
*g*	0	0.57	1.94

**Figure 14:**
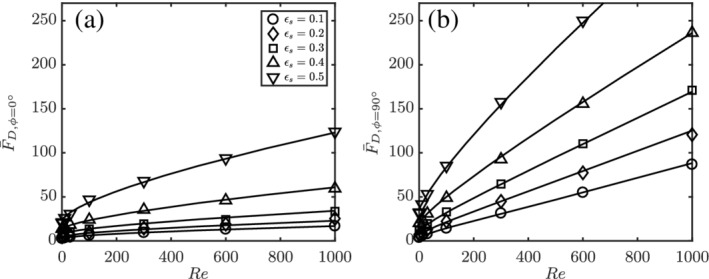
The averaged drag forces (a) ${\bar{F}}_{D,\phi =0^{{}^{\circ}}}$ and (b) ${\bar{F}}_{D,\phi =90^{{}^{\circ}}}$ at different Re and *ɛ*_s_. The markers indicate simulation data and the solid lines are corresponding fits

**Figure 15:**
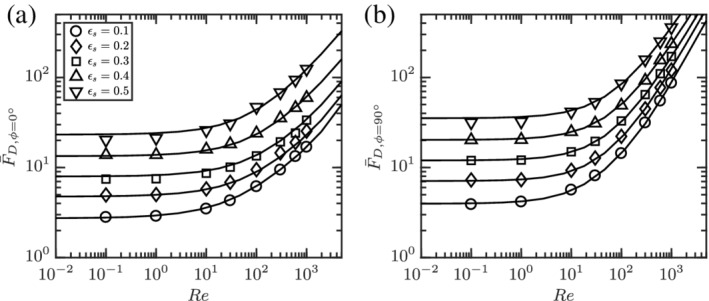
The fits for average (a) ${\bar{F}}_{D,\phi =0^{{}^{\circ}}}$ and (b) ${\bar{F}}_{D,\phi =90^{{}^{\circ}}}$ at different Re and *ɛ*_s_ beyond the simulated regimes of 0.1 ≤ Re ≤ 1,000. The markers indicate simulation data and the solid lines denote corresponding fits

The simulated data and corresponding fits are shown in Figure 14. The fits follow the physical limits beyond the Re range simulated as shown in Figure 15. In the Stokes flow limit, it can be observed that both *ϕ* = 0° and *ϕ* = 90° normalized drag becomes independent of Re. In the high Re limit, the normalized drag approaches a linear dependency on Re.

**Figure 16:**
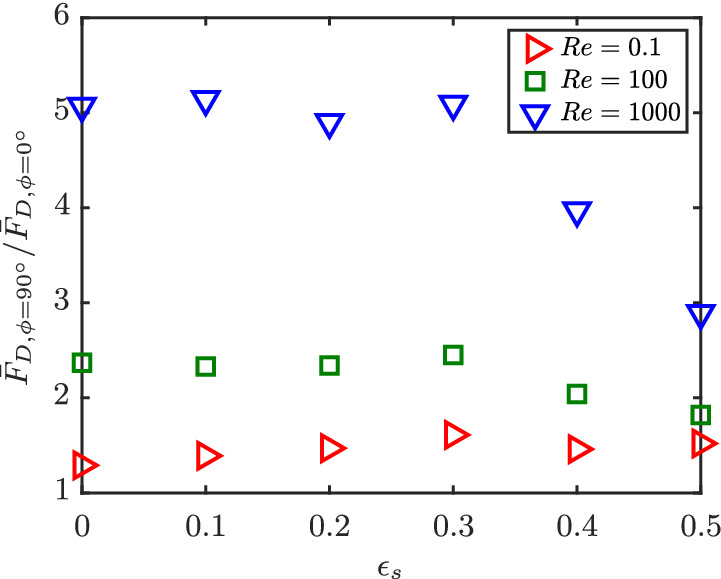
Ratio of average perpendicular to average parallel drag ${\bar{F}}_{D,\phi =90^{{}^{\circ}}}/{\bar{F}}_{D,\phi =0^{{}^{\circ}}}$ from simulations for different Re and *ɛ*_s_ [Color figure can be viewed at wileyonlinelibrary.com]

The ratio of average perpendicular to average parallel drag ${\bar{F}}_{D,\phi =90^{{}^{\circ}}}/{\bar{F}}_{D,\phi =0^{{}^{\circ}}}$ at different Re and *ɛ*_s_ is shown in Figure 16. For low Re (Re = 0.1), the ratio remains constant at a value a little larger than 1 for all *ɛ*_s_. The reason for this is that at low Re, the particles experience stronger viscous effects. The viscous drag reduces and pressure drag increases with increasing *ϕ* at low Re. The same has been confirmed for isolated particles^9^ and for a multiparticle system.^19^ The combined viscous and pressure drag components result in a drag ratio close to 1 for the considered spherocylinders at low Re. Due to inertial dominance at moderate and large Re (Re ≥ 100) we can observe a near constant drag ratio for solids volume fractions upto *ɛ*_s_ = 0.3 and a decrease in the ratio for *ɛ*_s_ > 0.3. Further, Figure 16 gives an indication that for very dense crowding, that is, at *ɛ*_s_ > 0.5, there is a possibility that ${\bar{F}}_{D,\phi =90^{{}^{\circ}}}/{\bar{F}}_{D,\phi =0^{{}^{\circ}}}$ tends back to approximately 1. Up to moderate crowding, although the flow is disturbed due to the presence of neighboring particles, there is sufficient interparticle space for flow to achieve uniformity. However, with increased particle crowding, there appear pronounced fluctuations in flow velocities (see also the section on flow histograms below), resulting in a reduced drag ratio at high *ɛ*_s_. This is an important finding because the traditional approach of Euler–Lagrangian simulations involve combining isolated nonspherical particle drag with the voidage effects based on sphere assemblies. This would result in a constant drag ratio ${\bar{F}}_{D,\phi =90^{{}^{\circ}}}/{\bar{F}}_{D,\phi =0^{{}^{\circ}}}$ independent of *ɛ*_s_. This in turn could affect Euler–Lagrangian simulation results, especially in predicting the minimum fluidization velocity as there exists a dense packing of particles. This mandates the need for the current work.

**Figure 17:**
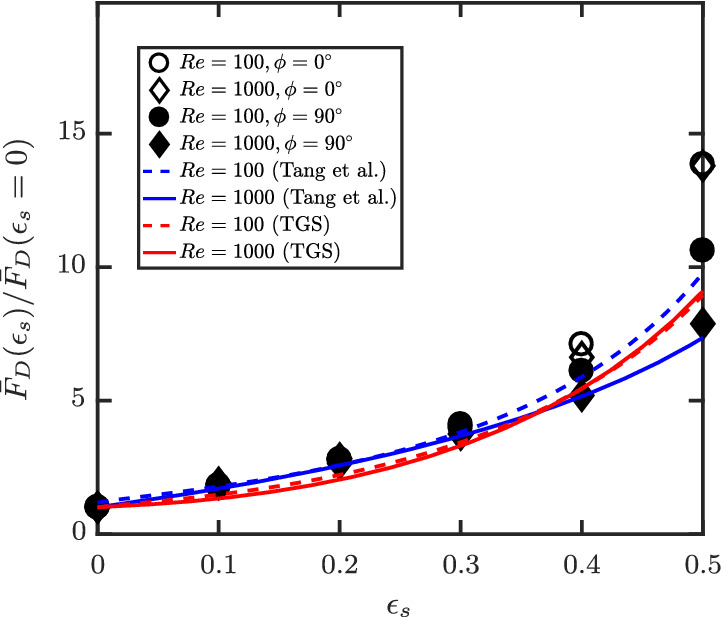
Voidage effect on average drag: ${\bar{F}}_{D}\left({ɛ}_{s}\right)/{\bar{F}}_{D}\left({ɛ}_{s}=0\right)$ for *ϕ* = 0° and *ϕ* = 90° in the inertial regimes as a function of *ɛ*_s_ for spherocylinders (this work, symbols), compared with voidage effect for spheres from literature. TGS denotes Tenneti et al^2^ [Color figure can be viewed at wileyonlinelibrary.com]

**Figure 18:**
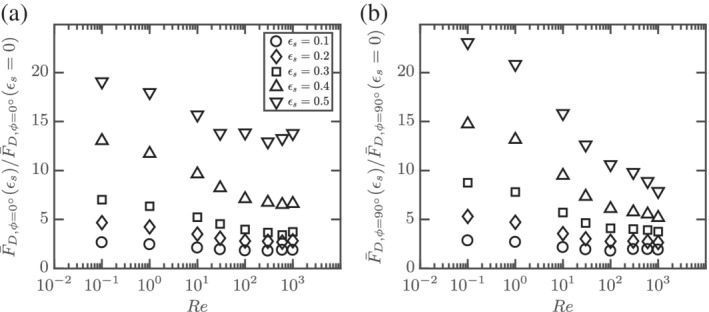
Voidage effect on average drag: ${\bar{F}}_{D,\phi =0^{{}^{\circ}}}\left({ɛ}_{s}\right)/{\bar{F}}_{D,\phi =0^{{}^{\circ}}}\left({ɛ}_{s}=0\right)$ and ${\bar{F}}_{D,\phi =90^{{}^{\circ}}}\left({ɛ}_{s}\right)/{\bar{F}}_{D,\phi =90^{{}^{\circ}}}\left({ɛ}_{s}=0\right)$ as a function of Re

Figure 17 shows a similar interesting observation: The scaling of the voidage effect ${\bar{F}}_{D}\left({ɛ}_{s}\right)/{\bar{F}}_{D}\left({ɛ}_{s}=0\right)$ in the inertial regime (high Re limit) is shape and orientation independent for *ɛ*_s_ ≤ 0.3. Here, we have normalized the average drag with respective isolated particle drag for different Re and *ϕ*. It can be observed that all the normalized points fall on a same trend until *ɛ*_s_ = 0.3. Similar normalized drag for spheres from Tang et al^3^ at Re = 100 and 1,000 also show the same trend until *ɛ*_s_ = 0.3. Here, we use the isolated sphere drag correlation of Schiller and Naumann^33^ for the normalization. The predictions of Tenneti et al^2^ for spheres do not follow the exact trend for the voidage effects as observed from Figure 17. It should be noted that Tenneti et al^2^ explored only until Re = 300 in their work and extrapolation to high Re may not apply. Therefore, the above discussion indicates that spherical drag correlations for the voidage effect, combined with isolated nonspherical particle drag correlations can be applied to dilute suspension simulations of nonspherical particles in the inertial regimes. For a given nonspherical particle, the effect of crowding (*ɛ*_s_) on ${\bar{F}}_{D}$ is different for different Re and *ϕ*. Figure 18 shows the voidage effect (average ${\bar{F}}_{D}$ normalized by the corresponding isolated particle drag) as a function of Re. It can be seen at low Re, the increase in drag due to crowding is comparable for both *ϕ* = 0° and *ϕ* = 90° at different *ɛ*_s_. At high Re, the increase in drag due to crowding with increasing *ɛ*_s_ is much stronger for *ϕ* = 0° compared to *ϕ* = 90°. This also explains further the reason for the observed reduction in average perpendicular to average parallel drag ratios with increasing *ɛ*_s_ in Figure 16. It also confirms that simple voidage effect correlations which only depend on *ɛ*_s_ and Re, such as the Richardson and Zaki law,^11^ cannot be used for highly nonspherical particles at higher *ɛ*_s_ and higher Re.

**Figure 19:**
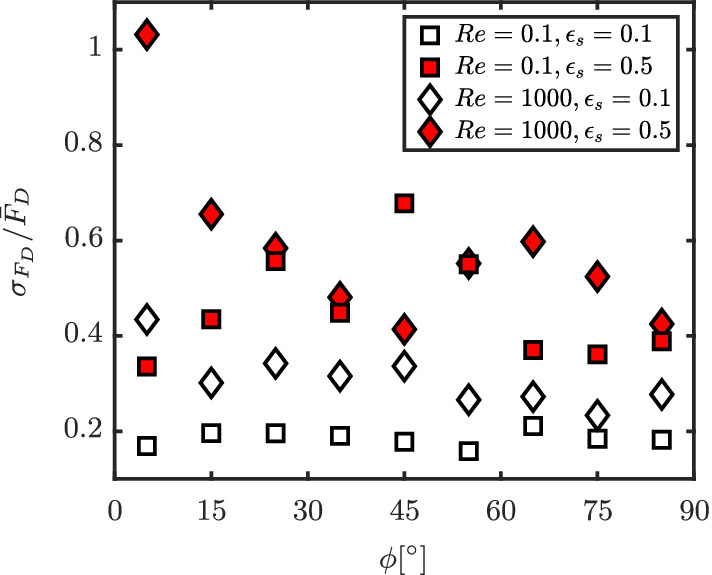
The standard deviations $\sigma_{F_{D}}$ of the distribution of individual drag values, normalized by the corresponding average ${\bar{F}}_{D}$ in different intervals of incident angle *ϕ*. Open symbols correspond to dilute configurations and filled symbols correspond to dense configurations [Color figure can be viewed at wileyonlinelibrary.com]

In the previous sections, we discussed the ${\bar{F}}_{D}$ averaged over all particles with similar *ϕ*. However, the distribution of *F*_D_ within a *ϕ* interval is itself also a function of both Re and *ɛ*_s_. The standard deviations of the distribution of drag measurements, normalized by the average ${\bar{F}}_{D}$ in the corresponding *ϕ* interval, are plotted in Figure 19. It is important that the standard deviations are normalized by the average ${\bar{F}}_{D}$ at respective *ϕ*, rather than against a single value, say ${\bar{F}}_{D,\phi =90^{{}^{\circ}}}$, for a given Re and *ɛ*_s_. This is because with increasing Re, the ratio ${\bar{F}}_{D,\phi =90^{{}^{\circ}}}/{\bar{F}}_{D,\phi =0^{{}^{\circ}}}$ increases, as shown in Figure 16. Therefore, using average ${\bar{F}}_{D,\phi =90^{{}^{\circ}}}$ for normalization will make the standard deviations at *ϕ* = 0° appear insignificant at large Re.

For dilute configurations (*ɛ*_s_ = 0.1), we clearly observe that increasing Re results in an increased $\sigma_{F_{D}}/{\bar{F}}_{D}$ at all *ϕ*. It should be noted that the absolute magnitudes of average ${\bar{F}}_{D}$ at Re = 1,000 are much larger than at Re = 0.1. Despite the normalization by these larger values, we observe increased standard deviations for higher Re. This is because at low Re, the viscous effects dominate, resulting in long‐range flow uniformity. Conversely, at high Re, the boundary layers are thinner and flow wakes are stronger. This results in high nonuniformity in the incoming flow on each particle, and thereby large fluctuations in the hydrodynamic forces. For dense particle configurations (*ɛ*_s_ = 0.5), it can be observed that $\sigma_{F_{D}}/{\bar{F}}_{D}$ increases relative to dilute conditions, with a higher standard deviation for higher Re. The reason for higher spread in *F*_D_ is due to the fact the particles locally encounter highly nonuniform incoming flows when there is more crowding.

### 4.2 Comparison with other literature

**Figure 20:**
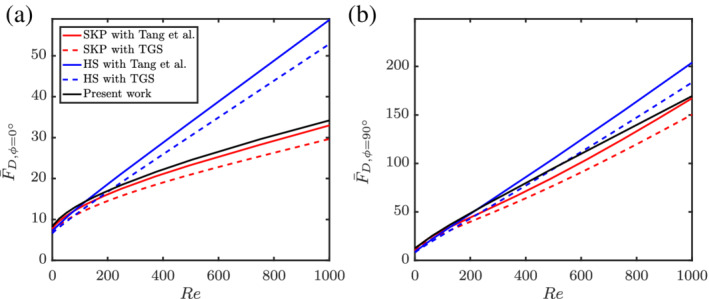
Comparison of average ${\bar{F}}_{D}$ for (a) *ϕ* = 0° and (b) *ϕ* = 90° for *ɛ*_s_ = 0.3. SKP denotes Sanjeevi et al,^4^ HS denotes Hölzer and Sommerfeld,^6^ and TGS denotes Tenneti et al.^2^ The solid black line is Equation (23) [Color figure can be viewed at wileyonlinelibrary.com]

**Figure 21:**
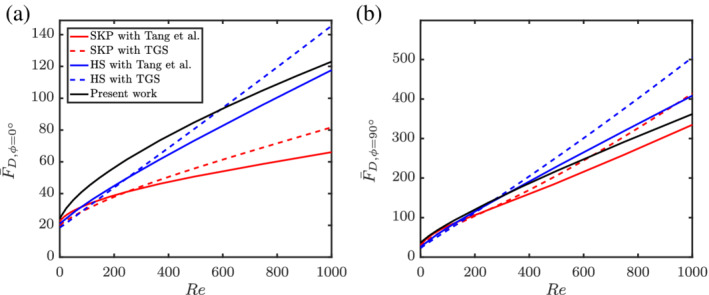
Comparison of average ${\bar{F}}_{D}$ for (*a*) *ϕ* = 0° and (*b*) *ϕ* = 90° for *ɛ*_s_ = 0.5. SKP denotes Sanjeevi et al,^4^ HS denotes Hölzer and Sommerfeld,^6^ and TGS denotes Tenneti et al.^2^ The solid black line is Equation (23) [Color figure can be viewed at wileyonlinelibrary.com]

Given the unavailability of multiparticle correlations for nonspherical particles at higher Reynolds numbers, we combine the available literature results on isolated nonspherical particles with voidage effects based on spheres. For this, we normalize the multiparticle drag of spheres with the isolated sphere Schiller and Naumann^33^ correlation and multiply with the isolated nonspherical particle drag. The results are shown in Figures 20 and 21 for *ɛ*_s_ = 0.3 and *ɛ*_s_ = 0.5, respectively. The isolated particles drag law used are SKP^4^ and HS.^6^ They are accordingly combined with the multiparticle effects of TGS^2^ and Tang et al^3^ for spheres. In the moderately crowded regime (*ɛ*_s_ = 0.3), our earlier suggestion of combining isolated nonspherical particle drag with multiparticle effects from spheres works well. For example, the combination of SKP with Tang et al^3^ follows nearly the same trend as that of the current work (Equation (23)). This can be observed for both *ϕ* = 0° and *ϕ* = 90°. However for dense regimes (*ɛ*_s_ = 0.5), it can be observed that the combination of SKP with Tang et al^3^ does not agree well with the present work for *ϕ* = 0°. At the same time, the combination with the HS^6^ isolated drag law seem to be closer to the current work for *ɛ*_s_ = 0.5. Such an agreement must be considered with care. The decent agreement occurs because HS possesses high drag values for *ϕ* = 0° (for *ɛ*_s_ = 0), in combination with a weak voidage effect for spheres. On the other hand, SKP with TGS or Tang et al^3^ show decent agreement with the present work for *ϕ* = 90°.

### 4.3 Lift

The normalized lift *F*_l, *ϕ*_ on a single elongated particle from Sanjeevi et al^4^ is given by

(27) Fl,ϕReϕ=Fl,isol⋅Sf,ϕ,with

(28) Fl,isolRe=b1Re+b2Reb3+b4Reb5Re24,and

(29) Sf,ϕReϕ=sinϕ1+b6Reb7cosϕ1+b8Reb9.

Here, *S*_f, *ϕ*_ is the scaling function dependent on Re and *ϕ*. The coefficients *b*_*i*_ are accordingly listed in the mentioned literature. In particular, the coefficients *b*_6_ to *b*_9_ describe the amount of skewness of the lift coefficient on a single elongated particle around *ϕ* = 45°. In the current work, we observe the same skewness for the multiparticle system at different Re. Therefore, we assume the term *S*_f, *ϕ*_ remains the same for the multiparticle system. The average lift ${\bar{F}}_{L}$ for a multiparticle system takes the following form:

(30) F¯L,ϕReɛsϕ=FL,magReɛs⋅Sf,ϕReϕ.

The functional form of *F*_L, mag_ (Re, *ɛ*_s_) remains similar to that of the drag and is given by

(31) FL,magReɛs=Fl,isolRe⋅1−ɛs2+Fɛsɛs+FRe,ɛsReɛs

with

(32) $$F_{{ɛ}_{s}}\left({ɛ}_{s}\right)=a\sqrt{{ɛ}_{s}}{\left(1-{ɛ}_{s}\right)}^{2}+\frac{b{ɛ}_{s}}{{\left(1-{ɛ}_{s}\right)}^{2}},\;\text{and}\;$$

(33) FRe,ɛsReɛs=Recɛsde1−ɛs+fɛs31−ɛs+gɛs1−ɛs2Re.

**Figure 22:**
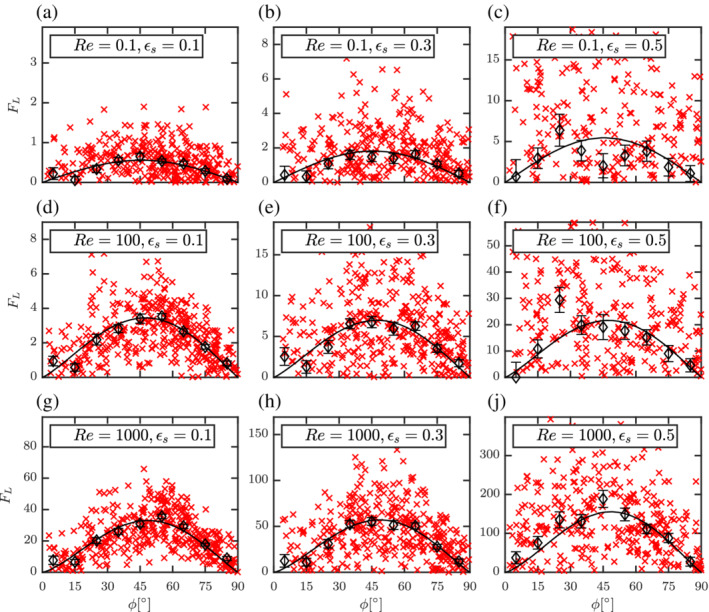
Distributions of lift forces *F*_L_ (**×**) with averages at regular *ϕ* intervals (*◊*) for different Re and *ɛ*_s_. The solid line denotes the ${\bar{F}}_{L,\phi }$ fit (Equation (30)). Each plot includes data from two independent simulations with a total 400 data points. It should be noted that the scales are different for each plot. The error bars indicate the standard error on the mean for each *ϕ* interval [Color figure can be viewed at wileyonlinelibrary.com]

The corresponding coefficients are given in Table 2. The proposed average lift correlation has around 5% average absolute deviation with respect to the averaged lift from simulations. The comparison of the average ${\bar{F}}_{L}$ from simulations and the proposed correlation is shown in Figure 22.

### 4.4 A simplified lift function

In our earlier works,^4^, ^9^ we have shown successfully that for isolated elongated particles, the relation between lift and drag in the Stokes flow regime can be successfully used for higher Re flows too. In other words, ${\bar{F}}_{L}$ at different *ϕ* can be computed as

(34) $${\bar{F}}_{L,\phi }=\left({\bar{F}}_{D,\phi =90^{{}^{\circ}}}-{\bar{F}}_{D,\phi =0^{{}^{\circ}}}\right)\sin\phi \cos\phi .$$

In this section, we show that Equation (34) is a reasonable approximation even for a multiparticle system. This implies that the scaling law is valid not only just for different Re but even for different *ɛ*_s_. Given a measured average ${\bar{F}}_{L}$ distribution from simulations at a given Re and *ɛ*_s_, the data can be fitted in a simple form as

(35) $${\bar{F}}_{L,\phi }=F_{L,\text{simple}}\sin\phi \cos\phi .$$

**Figure 23:**
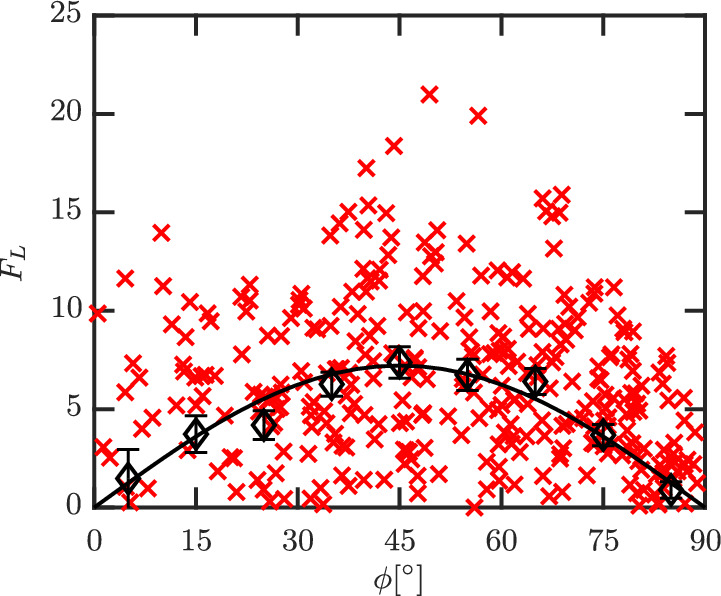
Distribution of *F*_L_ (**×**) for Re = 100 and *ɛ*_s_ = 0.3 with averages (*◊*) in regular *ϕ* intervals. The solid black line indicates the corresponding simple fit based on Equation (35). The fit includes data from two different simulations totalling 400 data points. The error bars indicate the standard error on the mean for each *ϕ* interval [Color figure can be viewed at wileyonlinelibrary.com]

**Figure 24:**
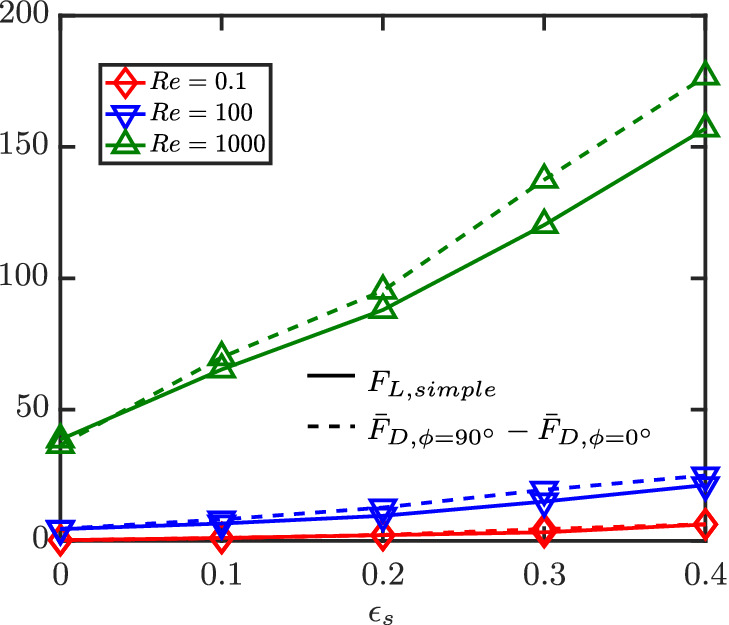
Comparison of ${\bar{F}}_{D,\phi =90^{{}^{\circ}}}-{\bar{F}}_{D,\phi =0^{{}^{\circ}}}$ with *F*_L, simple_ at different Re and *ɛ*_s_. The difference ${\bar{F}}_{D,\phi =90^{{}^{\circ}}}-{\bar{F}}_{D,\phi =0^{{}^{\circ}}}$ is based on averaged simulation data itself and not on the corresponding averaged ${\bar{F}}_{D}$ fits [Color figure can be viewed at wileyonlinelibrary.com]

Here, *F*_L, simple_ is a fit parameter that best describes the simulation data. An example for such a fit for Re = 100 and *ɛ*_s_ = 0.3 is given in Figure 23. The comparison of the Stokes regime lift law (Equation (34)) and our hypothesis (Equation (35)) is shown in Figure 24 and it can be observed that there is a good agreement. The highest absolute deviation observed between the equations is still less than 20% and average absolute deviation is around 12%. Therefore in Euler–Lagrangian simulations, in the absence of explicit lift data, Equation (34) can be applied to include the effects of lift with acceptable accuracy. This implies that in the often‐used approach of using Hölzer and Sommerfeld^6^ type drag correlations, combined with sphere‐based voidage effect correlations in Euler–Lagrangian simulations, one can also include lift effects based on Equation (34). In the following section, we will show the importance of including lift, as it is often of comparable magnitude to drag at high Re.

**Table 3:** Coefficients of the fits for *T*_P, mag_ (Equations 40, 41)

Coefficients	*T*_P, mag_
*a*	0.82
*b*	1.44
*c*	1.07
*d*	5.48
*e*	0.223

### 4.5 Importance of lift compared to drag

**Figure 25:**
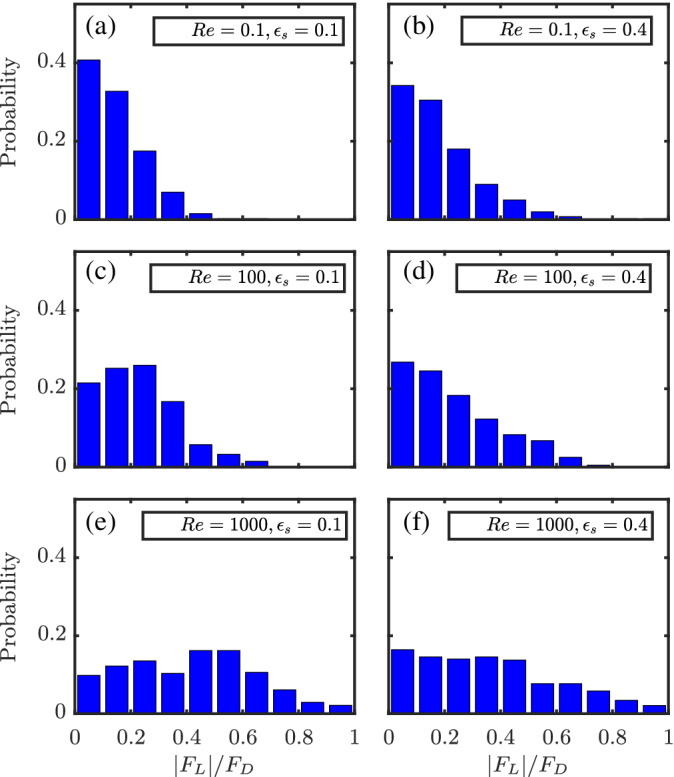
Distribution of lift force on individual particles normalized by corresponding drag force on each particle at different Re and *ɛ*_s_ [Color figure can be viewed at wileyonlinelibrary.com]

In Euler–Lagrangian simulations, the effect of lift forces is often neglected. This is because there is not much literature on nonspherical particle lift correlations. In this section, we analyse the magnitudes of lift compared to the drag on individual nonspherical particles at different Re and *ɛ*_s_. Figure 25 shows the distributions of the magnitude of the lift force relative to the drag force on each particle |*F*_L_|/*F*_D_. It can be observed that for Stokes flow (Re = 0.1), most particles experience lift which is about one order of magnitude smaller than the drag. However, for high Re (Re = 1,000), the distribution is much more wider spread and there are even some particles for which |*F*_L_|/*F*_D_ = 1. This emphasizes the need for including lift in Euler–Lagrangian simulations, especially while handling Geldart D particles, where the encountered particle Re is high. With increasing *ɛ*_s_, a different interesting observation can be made. In the low Re regime, increasing *ɛ*_s_ results in an increased probability of particles experiencing high lift magnitudes compared to the drag. On the contrary, at high Re (Re = 1,000), increasing *ɛ*_s_ results in the |*F*_L_|/*F*_D_ distribution skewing to the left. It should be noted that the highest *ɛ*_s_ shown in Figure 25 is *ɛ*_s_ = 0.4 as opposed to *ɛ*_s_ = 0.5, the highest *ɛ*_s_ explored. This is because random configurations are not possible for *ɛ*_s_ = 0.5. To ensure consistency, all results shown in Figure 25 are based on random configurations.

### 4.6 Torque

For an isolated nonspherical particle, the torque correlation^4^ is given by:

(36) Tp,ϕReϕ=Tp,isolRe⋅SϕReϕ,with

(37) Tp,isolRe=c1Rec2+c3Rec4Re32,and

(38) SϕReϕ=sinϕ1+c5Rec6cosϕ1+c7Rec8.

All coefficients can be found in our previous work.^4^ We note that for our particle geometry the isolated particle torque strictly increases with increasing Re (at least in the range of Re studied). It may be possible that at higher Re the torque will decrease again, as predicted by Khayat and Cox^34^ for slender bodies.

**Figure 26:**
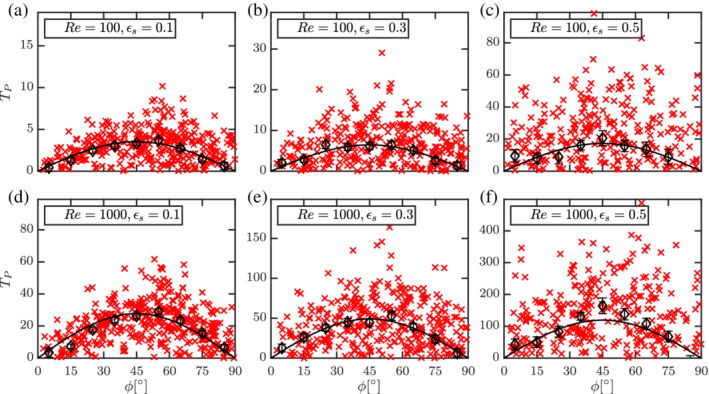
Distributions of *T*_P_ (**×**) with averages at regular *ϕ* intervals (*◊*) for different Re and *ɛ*_s_. The solid black line denotes *T*_P, *ϕ*_ fit (Equation (39)). Each plot includes data from two independent simulations with each containing 400 data points. It should be noted that the scales are different for each plot. The error bars indicate the standard error on the mean for each *ϕ* interval [Color figure can be viewed at wileyonlinelibrary.com]

The Re dependent skewness terms *c*_5_, *c*_6_, *c*_7_, *c*_8_ equal zero for an isolated spherocylinder resulting in a symmetric distribution for *ϕ* around 45°. Likewise, we also observe a near symmetric distribution of average torque at different Re and *ɛ*_s_ for the multiparticle configuration (see Figure 26). Unlike drag and lift, for an isolated nonspherical particle, the pitching torque vanishes for all *ϕ* in the Stokes flow regime. We observe the same for the multiparticle configuration. Therefore, the proposed correlation for the average torque ${\bar{T}}_{P}$ is applicable only in the inertial regime (10 < Re ≤ 1,000) and is given by

(39) T¯P,ϕReɛsϕ=TP,magReɛs⋅sinϕcosϕ,with

(40) TP,magReɛs=Tp,isolRe⋅1−ɛs2+TRe,ɛsReɛs.

The corresponding terms in the scaling are as follows (coefficients for the fit are given in Table 3):

(41) TRe,ɛsReɛs=Reaɛsbc1−ɛs+dɛs31−ɛs+eɛs1−ɛs2Re.

**Figure 27:**
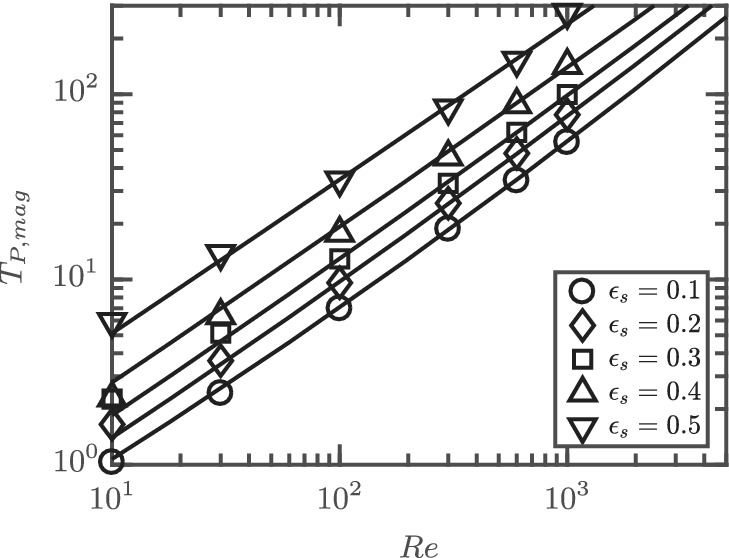
*T*_P, mag_ at different Re and *ɛ*_s_. The markers indicate simulation data and the solid line denotes fit at corresponding *ɛ*_s_

The average absolute deviation between Equation (39) and corresponding simulation data is 3%. It should be noted that *T*_P, mag_ in Equation (40) maps only the magnitude of the torque for different Re and *ɛ*_s_. The *ϕ* dependence is included separately with the *sine* and *cosine* terms. The comparison of *T*_P, mag_ and the corresponding simulation data are given in Figure 27. Given a symmetric form for ${\bar{T}}_{P,\phi }$, the *T*_P, mag_ is equal to twice the magnitude of $T_{P,\phi =45^{{}^{\circ}}}$ since sin*ϕ*cos*ϕ* = 1/2 at *ϕ* = 45°. From Figure 27, it can be observed that *T*_P, mag_ roughly follows the same power law dependence on Re for different *ɛ*_s_ because the slopes are similar. This is in contrast to the drag trends in Figure 15, where the trend starts from zero slope at low *ℜ* to a constant slope at high Re. This is caused by the fact that the average torque vanishes at low Re for all *ɛ*_s_.

### 4.7 Flow histograms

In the previous sections, we discussed the influence of the flow on the hydrodynamic forces and torques on the particles. The flow around particulate assemblies can also be viewed as flow through a porous medium. In this section, we discuss the results of the influence of the particles on the flow distribution.

**Figure 28:**
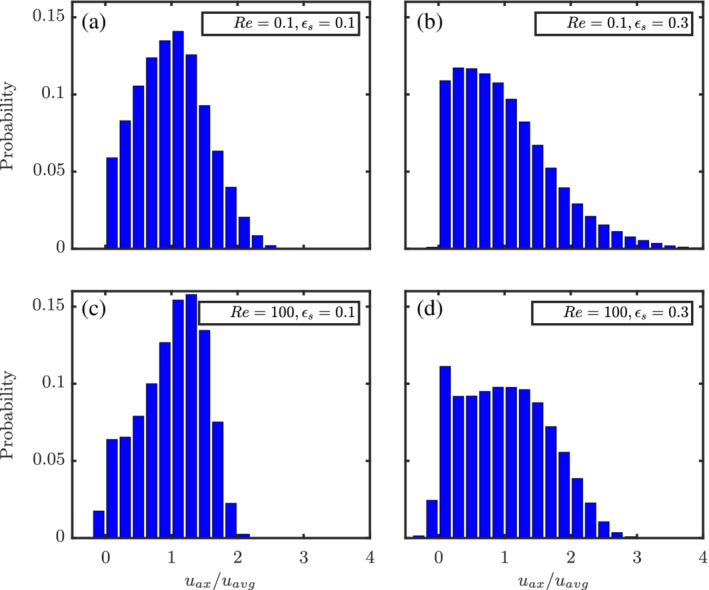
Axial‐velocity distributions at different Re and *ɛ*_s_ for a random configuration [Color figure can be viewed at wileyonlinelibrary.com]

The probability distributions of the normalized axial flow velocities (*u*_ax_/*u*_avg_) at different Re and *ɛ*_s_ for random configurations are given in Figure 28. Here, the normalization is done against the average axial velocity *u*_avg_ = *u*_s_/(1 − *ɛ*_s_) rather than the superficial velocity *u*_s_ to ensure a fair comparison for different *ɛ*_s_. Only the velocities of fluid cells are shown here and the zero velocities in the solid cells are ignored. It can be observed that with increasing Re, the spread of the velocity distribution becomes narrower. This can be simply attributed to the increased inertial effects and thinner boundary layers for increasing Re. Interestingly, the high Re flows also demonstrate some negative velocities corresponding to wake effects. With increasing *ɛ*_s_, the peaks of the distribution shift toward the left and the distribution itself spreads wider. This implies that the increased presence of particle surfaces at higher *ɛ*_s_ pulls the velocities of fluid cells toward zero (hence the left skewness). At the same time, the fluid accelerates in the bulk regions further removed from the particle surfaces resulting in increased velocities (and hence a wider distribution) to maintain the desired *u*_s_.

**Figure 29:**
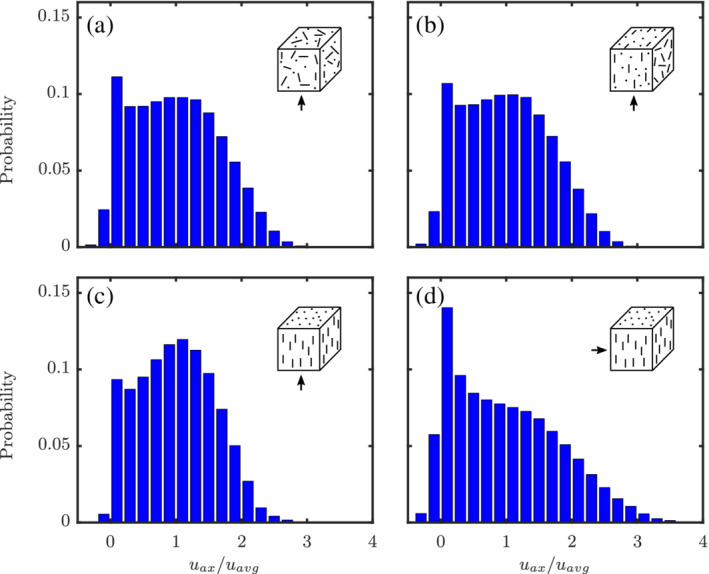
Axial‐velocity distributions for different configurations at Re = 100 and *ɛ*_s_ = 0.3 [Color figure can be viewed at wileyonlinelibrary.com]

It is also interesting to investigate the velocity distributions for different configurations for a given Re and *ɛ*_s_. The distributions of *u*_ax_/*u*_avg_ at Re = 100 and *ɛ*_s_ = 0.3 for different configurations are plotted in Figure 29. Given sufficient randomness of particles, as in random and planar random configurations (see Figure 29a,b), the velocity distributions are nearly identical. However, velocity distributions can be different for different configurations, as can be observed for the unidirectional configurations with flow parallel and perpendicular to the principal director (see Figure 29c, d). Among the different configurations shown, the unidirectional configuration with flow parallel to principal director has the least recirculation, as is evident from the least number of fluid cells with negative velocities (*u*_ax_/*u*_avg_ < 0). At the same time, the unidirectional configuration with flow perpendicular to principal director has the highest amount of recirculation. Overall, we can infer that there is no dependency between the configuration independence phenomenon and the flow velocity distribution of different configurations. The variation in forces at different incident angles *ϕ* is mainly arising from the pressure forces. The same can also be confirmed from the multiparticle work of He and Tafti,^19^ which is also in line with our finding for isolated nonspherical particles.^9^

## 5 CONCLUSION

The flow around static assemblies of axisymmetric, elongated, capsule‐like particles of aspect ratio 4 has been studied extensively using the multi‐relaxation‐time lattice Boltzmann method. The performed simulations are from the Stokes flow regime to high Re (0.1 ≤ Re ≤ 1,000) at different solids volume fraction *ɛ*_s_ (*ɛ*_s_ ≤ 0.5) and different mutual orientations of particles.

In general, average forces on random assemblies of spheres are only dependent on Re and *ɛ*_s_. Considering the nonspherical nature of the particles, we proposed four additional parameters to describe the flow problem: two to parametrize the mutual orientation of the nonspherical particles (*S*_1_ and *S*_2_) and two to represent the polar and azimuthal angles (*α* and *β*) of the averaged flow velocity with respect to the configuration. For this, we have developed different static particle configurations using Monte‐Carlo simulations. In the process, the configurations are biased to the desired amount of nematic or biaxial orientational order with the use of a Maier‐Saupe potential. The flow simulations indicate that the average particle forces are configuration independent, at least for *ɛ*_s_ ≤ 0.4, implying that the four additional parameters do not influence the results. The only important parameter representing orientation dependence is the incident angle *ϕ* of individual particles with respect to the average flow direction. We expect this result applies more generally to sufficiently elongated axisymmetric particles.

The configuration independence greatly simplifies the parameter space to be explored from 6 to 3 dimensions, namely Re, *ɛ*_s_, and *ϕ*. Of the three, the simulations are set up for only two parameters: Re and *ɛ*_s_. Given a sufficiently random particle configuration, different incident angles *ϕ* are covered automatically. Another interesting result from the current work is that our previous finding of sine‐squared scaling of drag for isolated nonspherical particles^9^ applies also to static monodisperse assemblies containing axisymmetric, elongated particles. In other words, given a Re and *ɛ*_s_, the average drag on the subset of particles oriented at an incident angle *ϕ* with respect to the superficial flow velocity can be described with the knowledge of average drag at *ϕ* = 0° and *ϕ* = 90° alone. This information can be used in a packed bed to determine the pressure drop across the bed with the knowledge of *ϕ* distribution alone. In a multiparticle configuration, also the average lift on a particle at an incident angle *ϕ* can be computed with good accuracy using the average drag at *ϕ* = 0° and *ϕ* = 90°, as in our previous work on isolated nonspherical particles. Having identified the dependent parameters, we proposed correlations for average drag, lift, and torque for a multiparticle configuration of aspect ratio 4 spherocylinders. During the process, we used correlations for isolated nonspherical particles and extended them to the multiparticle systems.

We have also explored the validity of the conventional approach of combining known correlations for isolated nonspherical particle drag with correlations for voidage effects based on sphere packings. We observe that in the dilute and intermediate *ɛ*_s_ regimes (*ɛ*_s_ ≤ 0.3), the influence of *ɛ*_s_ is nearly shape independent. This implies that the above conventional approach can safely be used to mimic flow around assemblies of nonspherical particles upto intermediate *ɛ*_s_. However, for denser regimes, there is a need for multiparticle simulations and hence the need for this work. In the inertial regimes, the ratios of average drag at *ϕ* = 90° and *ϕ* = 0° (${\bar{F}}_{D,\phi =90^{{}^{\circ}}}/{\bar{F}}_{D,\phi =0^{{}^{\circ}}}$) are nearly constant until *ɛ* ≤ 0.3 and then decrease with increasing *ɛ*_s_. This further proves that the conventional approach is not valid for dense regimes. In the process, we have analysed the flow‐velocity distribution as function of Re and *ɛ*_s_. Likewise, the influence of different particle configurations on the flow velocities have also been analysed.

Although individual forces on particles in a multiparticle environment are scattered around the reported averages, in Euler–Lagrangian simulations of dense particle flows the most important determining factor for the overall solids motion is the average force on a cluster of particles. This is the reason why CFD‐DEM simulations are so succesfull, for instance in predicting the dynamics of fluidized beds containing spherical particles, even though in reality the drag forces on individual particles are hugely scattered around the average force at a given mean voidage and Reynolds number.

Overall, this work provides a recipe to parametrize the average drag, lift, and torque experienced by monodisperse, axisymmetric, elongated particles in multiparticle environment. To the best of the authors' knowledge, there exists no work which parametrizes the drag, lift, and torque for nonspherical particles in a multiparticle environment. Generally, lift and torque are ignored in large‐scale Euler–Lagrangian simulations. The proposed accurate drag, lift, and torque correlations enable future Euler–Lagrangian simulations to be performed with more realistic physics for these particles of aspect ratio 4.
